# Crude childhood vaccination coverage in West Africa: Trends and predictors of completeness

**DOI:** 10.12688/wellcomeopenres.10690.1

**Published:** 2017-02-15

**Authors:** Jacob S. Kazungu, Ifedayo M.O. Adetifa

**Affiliations:** 1 Epidemiology and Demography Department, KEMRI-Wellcome Trust Research Programme, Centre for Geographic Medicine Research (Coast), Kilifi, Kenya; 2 Department of Public Health, Pwani University, Kilifi, Kenya; 3 Department of Infectious Disease Epidemiology, London School of Hygiene and Tropical Medicine, London, UK; 4 College of Medicine, University of Lagos, Lagos, Nigeria

**Keywords:** Vaccination coverage, dropout rates, trends and predictors, fully immunised vhild, West Africa

## Abstract

**Background**: Africa has the lowest childhood vaccination coverage worldwide. If the full benefits of childhood vaccination programmes are to be enjoyed in sub-Saharan Africa, all countries need to improve on vaccine delivery to achieve and sustain high coverage. In this paper, we review trends in vaccination coverage, dropouts between vaccine doses and explored the country-specific predictors of complete vaccination in West Africa. **Methods**: We utilized datasets from the Demographic and Health Surveys Program, available for Benin, Burkina Faso, The Gambia, Ghana, Guinea, Cote d’Ivoire, Liberia, Mali, Niger, Nigeria, Senegal, Sierra Leone and Togo, to obtain coverage for Bacillus Calmette-Guerin, polio, measles, and diphtheria, pertussis and tetanus (DPT) vaccines in children aged 12 – 23 months. We also calculated the DPT1-to-DPT3 and DPT1-to-measles dropouts, and proportions of the fully immunised child (FIC). Factors predictive of FIC were explored using Chi-squared tests and multivariable logistic regression. **Results**: Overall, there was a trend of increasing vaccination coverage. The proportion of FIC varied significantly by country (range 24.1-81.4%, mean 49%). DPT1-to-DPT3 dropout was high (range 5.1% -33.9%, mean 16.3%). Similarly, DPT1-measles dropout exceeded 10% in all but four countries. Although no single risk factor was consistently associated with FIC across these countries, maternal education, delivery in a health facility, possessing a vaccine card and a recent post delivery visit to a health facility were the key predictors of complete vaccination. **Conclusions**: The low numbers of fully immunised children and high dropout between vaccine doses highlights weaknesses and the need to strengthen the healthcare and routine immunization delivery systems in this region. Country-specific correlates of complete vaccination should be explored further to identify interventions required to increase vaccination coverage. Despite the promise of an increasing trend in vaccination coverage in West African countries, more effort is required to attain and maintain global vaccination coverage targets.

## Background

Over the last half-century or more, vaccination, as one of the most cost effective public health interventions ever, has been key to reducing child morbidity and mortality worldwide ^1^. Vaccination is essential to preventing target diseases of interest ^2^. Consequently, coverage above the minimum thresholds for the target disease or global targets is required to enjoy the full benefits of vaccination ^1, 3, 4^. Vaccination coverage is a performance indicator of immunisation programmes, which is also used to track global and national progress in the control of vaccine-preventable diseases (VPD). It is also an eligibility criterion for funding in many low and middle-income countries (LMICs) ^5^. 

With massive global and national investments in vaccination programmes, there have been significant improvements in global childhood vaccination coverage. For example the proportion of children who received the third dose of diphtheria-tetanus-pertussis vaccine (DTP3) by 12 months of age increased from 5% in 1974 to 86% in 2015 ( http://www.who.int/immunization/monitoring_surveillance/who-immuniz-2015.pdf?ua=1) ^6^. Unfortunately, DTP3 coverage has since stagnated at 85% since 2010 and many LMICs in sub-Saharan Africa (sSA), Eastern Mediterranean and South East Asia Regions of the world have not attained the recommended targets of 90% national vaccination coverage and <10% dropout between vaccine doses. ( http://www.who.int/immunization/global_vaccine_action_plan/en/ and http://www.who.int/immunization/monitoring_surveillance/who-immuniz-2015.pdf?ua=1)

These global data masks the widely variable vaccination coverage in LMICs, particularly in sSA countries that only attained 76% DTP3 coverage in 2015 ^6^. Besides, there are also regional differences in DTP3 coverage of 69% in West and Central compared to 79% in Eastern and Southern Africa ( http://data.unicef.org/corecode/uploads/document6/uploaded_pdfs/corecode/Immunization_Summary_2012_Eng_40) . When the fully immunised child is considered, less than half of children in its Eastern region are fully immunised ^7^. There are also widely varying vaccination coverage in countries within a region, for example overall DTP3 coverage ranges from 56% in Nigeria to 97% in The Gambia ( http://apps.who.int/immunization_monitoring/globalsummary/countries?countrycriteria[country][]=GMB). Yet achieving and maintaining high vaccination coverage could potentially avert millions of VPD-related deaths in children and yield into an estimated $63 billion in savings during the decade 2011 – 2020 ^8^. Good quality vaccination data is required to understand inequities in access to vaccines. Most vaccination coverage estimates in LMICs are from administrative reports that tend to overestimate coverage, due to errors in the number of vaccine doses administered and/or invalid assumptions about the size of the target population of children.

DPT3 for children sampled between 12 and 23 months is the principal surrogate measure of vaccination coverage and performance of national immunization programmes. However, the proportions of fully immunised children are often considered better indicators of the full benefits of immunisation compared to DPT3 within countries ^9^.

Using datasets from the Demographic and Health Surveys Program (DHS: https://dhsprogram.com/), we conducted a comprehensive review of the trends in vaccination coverage, dropouts between vaccine doses and country-specific predictors of a fully immunised child (FIC) in the West African region.

## Methods

### Study area and population

This study utilized datasets from DHS conducted in 13 West African countries: Benin, Burkina Faso, Cote d’Ivoire, The Gambia, Ghana, Guinea, Liberia, Mali, Niger, Nigeria, Senegal, Sierra Leone and Togo. DHS methodology encompasses a two-stage cluster sample design that produces unique, consistent, and nationally representative data that are comparable across countries ^10^. While these DHS datasets are not primarily carried out to collect vaccination data, they incorporate a questionnaire for women of reproductive age (15–49 years) for maternal and child health (including immunisation) in relation to all births within the preceding five years ^5^. DHS survey interviewers obtain immunization information from vaccine cards and/or mother’s/respondent’s recall.

For countries with multiple datasets between 2000 and 2013, we assessed trends in vaccination coverage using their two most recent standard DHS datasets, as follows: Benin (2006 and 2011–12); Burkina Faso (2003 and 2010); Ghana (2003 and 2008); Guinea (2005 and 2012); Liberia (2007 and 2013); Mali (2006 and 2012–13); Niger (2006 and 2012); Nigeria (2008 and 2013); Senegal (2005 and 2010–11) and Sierra Leone (2008 and 2013). The rest of the analyses to calculate dropouts and determine the predictors of a FIC included countries with single datasets (Cote d'Ivoire 2011–12, The Gambia 2013, and Togo 2013–14) and the most recent dataset for those countries with multiple datasets.

### Data management and analysis

We followed the widely recommended strategy for measuring complete vaccination status by restricting our datasets to children aged 12–23 months and dropping all children that had passed away by the date of interviews ^7, 11, 12^.

Our primary outcome was the fully immunised child (FIC). A FIC was defined as having received at birth or first contact, a dose of Bacille Calmette-Guérin vaccine (BCG), a 3-dose course of the diphtheria, pertussis and tetanus combination vaccine (DPT), and oral polio vaccine (OPV; given at 6, 10 and 14 weeks or at least four weeks apart) and a dose of measles-containing vaccine (MCV1; administered at 9 months), as reported by vaccine card or caregiver recall ^9, 13^. Other outcomes of interest in this study included access and utilization to immunization services. Good access was defined as having a DPT1 coverage of >80%, whereas a good utilization was defined as a DPT1-to-DPT3 dropout <10% ^14^.

All statistical analyses were performed using STATA software, version 13.1 (StataCorp, Lakeway Drive, College Station, TX, USA). In descriptive analysis, we reported the proportions of FIC and those who received each vaccine dose by country, as well as the percentage DPT1-to-DPT3 and DPT1-to-MCV1 dropout ^15^.

Chi-square tests were utilized in univariate analyses to examine associations between FIC and possible risk factors. The risk factors considered were: maternal age, maternal education, gender, religion, place of delivery, marital status, distance from home to nearest health facility, possession of a vaccine card, number of siblings, birth order, socio-economic status, rural or urban residence, and whether the child received a check-up within two months of birth ^7, 13, 16– 21^. Following this, we constructed multivariable logistic regression models within each country to examine the correlates of FIC. All factors identified at 10% significance (P-value <0.10) in univariate analysis were incorporated into the model. Before fitting the model, we assessed for potential collinearity using the Pearson’s R correlation coefficient (r > =0.8), and retained strongly correlated variables as suggested in the literature ^22, 23^.

To account for the complex DHS survey design, the *svyset* command in STATA was used to apply inverse probability weights ( http://www.ats.ucla.edu/stat/stata/faq/svy_introsurvey.htm). Adjusted odds ratios (AORs) and 95% confidence intervals are reported at a 5% significance level.

### Ethics statement

Ethical approval was not required for this study because it used anonymised DHS data. DHS surveys are conducted only after approvals have been given by the ICF International Institutional Review Board (IRB) and country IRBs for country-specific DHS survey protocols. In addition, written informed consent is obtained from each survey participant ( http://www.dhsprogram.com/What-We-Do/Protecting-the-Privacy-of-DHS-Survey-Respondents.cfm. The aggregate data utilised in this study was made freely available by DHS after after a simple registration process on their website ( http://www.dhsprogram.com/data/new-user-registration.cfm), which includes providing an explaination for the need for the datasets and planned analyses.

## Results

### Coverage by vaccine and proportions of the FIC

As shown in Table 1, coverage for each vaccine also varied by country. For BCG, it ranged from 50.6% in Nigeria to 98.4% in The Gambia, with only seven countries attaining the 90% Global Vaccine Action Plan target. Similarly, DPT1-to-DPT3 coverage remained low in a number of countries, with DPT3 coverage not reaching the 90% target in all countries. Despite similarly low OPV1-to-OPV3 coverage proportions, a sharp difference in DPT3 and OPV3 was observed in Nigeria – 38.0% and 53.3%, respectively. For measles, the 90% target was only achieved in Ghana (90.2%).

**Table 1.** Percentage coverage for each vaccine in children aged 12–23 months across selected West African countries. BCG, Bacille Calmette-Guérin vaccine; DPT, diphtheria, pertussis and tetanus; OPV, oral polio vaccine; FIC, fully immunised child.

Country	Survey year	BCG	DPT1	DPT2	DPT3	OPV1	OPV2	OPV3	Measles	FIC
**Benin**	**2011–12**	86.0	69.0	65.6	58.7	81.1	75.3	53.1	67.8	35.0
**Burkina Faso**	**2010**	96.4	94.3	92.7	89.5	97.3	95.5	90.2	87.3	81.2
**Cote d'Ivoire**	**2011–12**	83.0	77.4	71.6	63.8	91.1	83.7	69.0	63.9	49.6
**Gambia**	**2013**	98.4	94.1	93.3	87.8	96.6	94.3	88.7	87.3	72.5
**Ghana**	**2008**	95.7	97.5	95.5	89.3	96.7	94.3	86.8	90.2	78.8
**Guinea**	**2012**	82.3	75.5	62.3	49.9	84.2	71.4	51.1	61.4	36.1
**Liberia**	**2013**	93.8	91.3	82.0	71.5	95.4	86.8	70.3	74.1	54.6
**Mali**	**2012–13**	78.3	74.2	68.8	58.6	77.5	70.3	45.2	66.6	33.9
**Niger**	**2012**	83.5	85.2	78.4	68.2	91.2	84.6	75.3	68.0	51.0
**Nigeria**	**2013**	50.6	50.0	45.1	38.0	75.8	69.4	53.3	41.4	24.1
**Senegal**	**2010–11**	93.8	92.9	89.9	81.8	93.8	89.9	72.1	81.1	60.6
**Sierra Leone**	**2013**	95.4	93.0	88.2	78.1	93.5	88.3	77.9	77.9	67.0
**Togo**	**2013–14**	95.0	92.9	89.1	82.5	93.7	88.9	73.8	74.1	60.5

The proportions of the FIC varied across the 13 countries ranging from 24.1% in Nigeria to 81.2% in Burkina Faso ( Table 2). These proportions were significantly different (p< 0.01). Burkina Faso also had the lowest numbers of partially vaccinated children. The highest proportions of completely unvaccinated and partially vaccinated children were seen in Nigeria, whereas Ghana had the lowest proportion of unvaccinated children.

**Table 2.** Proportions of the Fully Immunized Child ^*^ in children aged 12–23 months across selected West Africa countries.

	Benin	Burkina Faso	Cote d'Ivoire	Gambia	Ghana	Guinea	Liberia	Mali	Niger	Nigeria	Senegal	Sierra Leone	Togo	p- value ^2^
	2011–12	2010	2011–12	2013	2008	2012	2013	2012–13	2012	2013	2010–11	2013	2013–14	
	N=2535 ^1^	N=2822 ^1^	N=1432 ^1^	N=1660 ^1^	N=552 ^1^	N=1296 ^1^	N=1272 ^1^	N=1846 ^1^	N=2275 ^1^	N=5900 ^1^	N=2169 ^1^	N=2169 ^1^	N=1395 ^1^	
	%	%	%	%	%	%	%	%	%	%	%	%	%	
**Fully** **immunized**	35.0	81.2	49.6	72.5	78.8	36.1	54.6	33.9	51.0	24.1	60.6	67.0	60.5	<0.01
**Partially** **immunized**	53.3	17.0	45.5	26.3	20.2	53.0	43.8	49.0	44.4	54.9	36.2	29.5	36.0	
**Not** **immunized**	11.7	1.8	4.9	1.3	1.0	10.9	1.6	17.1	4.6	21.0	3.2	3.5	3.5	

^*^Complete vaccination coverage as recommended by World Health Organisation (WHO) (a dose of BCG, 3 doses of polio, 3 doses of DPT and a dose of measles vaccine) ^1^Weighted values ^2^P-values corresponding *x* ^2^ test used to determine statistically significant differences by country in the percentages of children ages 12–23 months, who received complete vaccination according to WHO recommendation.

### Trend in vaccination coverage

Across all 10 countries, there was an overall increase in vaccination coverage for all vaccines, but country-specific coverage actually declined by over 25% in Benin and Mali when their last two DHS datasets are compared (see Table 3). BCG coverage increased in all countries except in Benin. In particular, Burkina Faso, Liberia, and Niger achieved substantial improvements in coverage of this vaccine.

**Table 3.** Trends in vaccination coverage in children aged 12–23 months across selected countries in West Africa.

Country	Benin		Burkina Faso		Ghana		Guinea		Liberia		Mali		Niger		Nigeria		Senegal		Sierra Leone	
Survey Year	2006	2011–12	2003	2010	2003	2008	2005	2012	2007	2013	2006	2012–13	2006	2012	2008	2013	2005	2010–11	2008	2013
**Percentage** **change**	**%**		**%**		**%**		**%**		**%**		**%**		**%**		**%**		**%**		**%**	
**BCG**	-2.5		21.1		5.7		3.5		22.1		2.8		31.7		2.8		2.5		17.5	
**DPT1**	-17.6		25.2		7.7		-1.8		22.1		-10.2		47.4		-2.7		-0.3		22.7	
**DPT2**	-14.8		38.2		8.2		42.5		-4.9		-9.8		62.7		1.3		1.7		27.3	
**DPT3**	-12.6		58.4		12.3		-2.7		43.9		-14.0		72.7		7.0		4.7		29.3	
**OPV1**	-7.9		13.8		4.5		2.2		15.9		-8.5		17.7		13.3		0.1		25.3	
**OPV2**	-5.6		28.9		6.6		2.4		20.9		-8.5		19.7		21.8		4.1		30.0	
**OPV3**	-17.5		53.9		9.5		1.0		44.4		-27.3		34.0		38.1		-1.1		57.7	
**MCV1**	11.1		57.0		9.5		22.8		19.5		-2.2		45.6		0.7		11.6		32.3	
**Not** **Vaccinated**	69.6		-81.1		-80.0		-22.1		-86.9		35.7		-72.1		-27.6		-15.8		-78.3	
**Received all** **8 vaccine** **doses**	-25.4		90.2		15.4		-2.4		45.2		-28.3		78.9		9.5		5.0		73.1	

DPT1 coverage declined in five countries - Benin, Guinea, Mali, Nigeria and Senegal - while DPT2 coverage increased in all countries except Benin, Liberia, and Mali. The biggest gains in DPT3 coverage was seen in Niger, Burkina Faso, Liberia and Sierra Leone. For DPT3, none of the countries attained the 90% target, with three countries recording a decline in coverage (see Table 1 and Table 3).

For the polio vaccines, OPV1, OPV2, and OPV3 coverage increased in all countries except for Benin and Mali, where there was a 27% reduction in coverage. OPV3 coverage also declined slightly in Senegal. For OPV3, the biggest gains in coverage were seen in Sierra Leone, Burkina Faso, Liberia, Nigeria and Niger.

Despite a decline in coverage of all other vaccines in Benin, MCV1 coverage actually increased, which was also seen in all other countries except Mali. The biggest gains in MCV1 coverage were seen in Burkina Faso, Niger and Sierra Leone. Recommended MCV1 coverage targets were not met in any of these West African countries, except for Ghana ( Table 1).

Across all countries, there were major reductions in the number of unvaccinated children, ranging from 22–81% reduction, except in Benin and Mali where they increased by 70% and 36%, respectively. Similarly, the proportions of the FIC increased in most countries, except Benin, Guinea, and Mali.

### Dropout proportions

***DPT1-to-DPT3.*** The proportion of DPT1-to-DPT3 dropouts varied across countries ranging from 5.1% in Burkina Faso to a high of 33.9% in Guinea. All except three countries - Burkina Faso, Gambia, and Ghana - had high DPT-1-to-DPT-3 dropouts (i.e. >10%) ^24, 25^, indicating a region-wide problem with utilisation of immunisation services ( Table 4). Based on their low DPT1 coverage (<80%), five out of 13 countries also had poor access to immunisation services ^24, 25^. Three out of 13 countries reported high DPT1 coverage (>80%), a correlate of good access to immunisation services, and a low DPT1-to-DPT3 dropout (<10%), which is an indication of good utilization of these services.

**Table 4.** Proportion of dropouts beween DPT1 and DPT3 in West African children aged 12–23 months by country.

Country	DPT3 vaccinated	DPT1 vaccinated	Drop out	Dropout %	Access	Utilization	Problem Type
**Benin**	58.7	69.0	10.3	14.9	Poor	Poor	4
**Burkina** **Faso**	89.5	94.3	4.8	5.1	Good	Good	1
**Cote** **d'Ivoire**	63.8	77.4	13.6	17.6	Poor	Poor	4
**Gambia**	87.8	94.1	6.3	6.7	Good	Good	1
**Ghana**	89.3	97.5	8.2	8.4	Good	Good	1
**Guinea**	49.9	75.5	25.6	33.9	Poor	Poor	4
**Liberia**	71.5	91.3	19.8	21.7	Good	Poor	2
**Mali**	58.6	74.2	15.6	21.0	Poor	Poor	4
**Niger**	68.2	85.2	17.0	20.0	Good	Poor	2
**Nigeria**	38.0	50.0	12.0	24.0	Poor	Poor	4
**Senegal**	81.8	92.9	11.1	11.9	Good	Poor	2
**Sierra** **Leone**	78.1	93.0	14.9	16.0	Good	Poor	2
**Togo**	82.5	92.9	10.4	11.2	Good	Poor	2

Drop out = DPT1 – DPT3 Dropout % = (Dropout/DPT1)* 100 1 = Drop-out rates are low (<10%)= good utilization & DPT1 coverage is high (>=80%)= good access 2 = Drop-out rates are high (>=10%)= poor utilization & DPT1 coverage is high (>=80%)= good access 3 = Drop-out rates are low (<10%)= good utilization & DPT1 coverage is low (<80%)= poor access 4 = Drop-out rates are high (>=10%)= poor utilization & DPT1 coverage is low (<80%)= poor access

**Figure 1.**
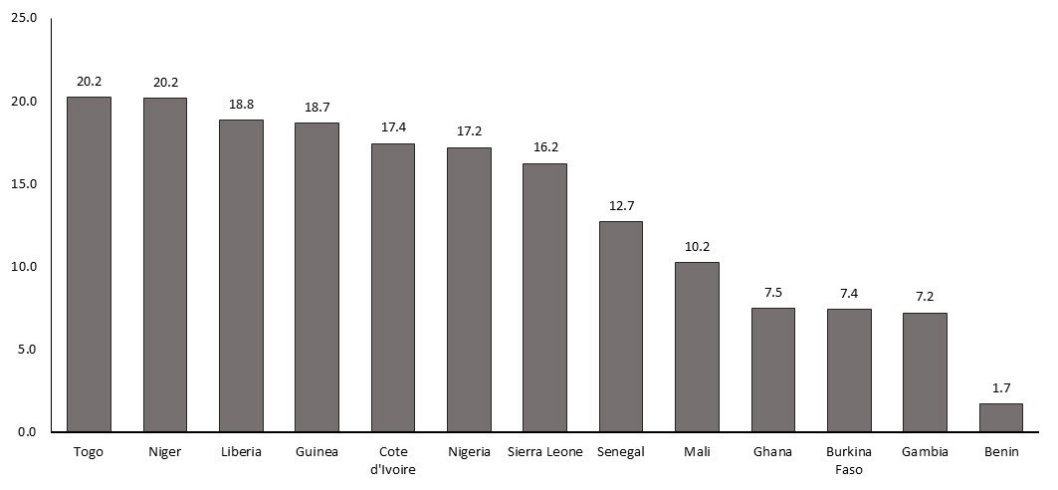
Proportion of DPT1-to-MCV1 dropouts among children aged 12–23 months in selected West African countries.

***DPT1-to-MCV1.*** Given the DPT1-to-MCV1 dropout of <10% in Benin, Burkina Faso, Ghana and The Gambia, children in these countries were more likely to have received all the recommended vaccines by one year of age compared to children from all other countries reviewed ( Figure 1).

### Predictors of complete vaccination status

In univariate analysis, possession of a vaccine card was associated with increased likelihood of FIC status in all countries. In addition, delivery at a hospital/health facility, and attendance of a well-baby clinic for review/check-up within two months of birth were related to complete vaccination in all countries, except Gambia and Ghana.

Although many countries shared the same predictors or risk factors for complete vaccination in multivariable regression models, the results were still quite variable as shown in the AORs presented in Table 5. Delivery at a health facility remained a significant predictor of a FIC, such that children born at a health facility were 1.5–2.3 times more likely to be fully immunised compared to those born at home in Benin, Burkina Faso, Cote d’Ivoire, Guinea, and Nigeria.

**Table 5.** Predictors of complete vaccination status according to WHO recommendations for FIC status in West African children aged 12–23 months.

	Benin	Burkina Faso	Cote d'Ivoire	The Gambia	Ghana	Guinea	Liberia	Mali	Niger	Nigeria	Senegal	Sierra Leone	Togo
**Visit within 2** **months**	1.5 (1.2, 1.8)*	1.7 (1.2, 2.3)*	1.6 (1.1, 2.3)*	-	-	1.6 (1.1, 2.3)*	-	1.5 (1.1, 1.9)*	2.1 (1.6, 2.7)*	1.4 (1.2, 1.8)*	1.7 (1.3, 2.3)*	-	1.5 (1.0, 2.1)*
**Birth order**												
**1 ^st^**	-	-	-	-	-	-	Ref	-	-	Ref	-	-	Ref
**2 ^nd^–3 ^rd^**	-	-	-	-	-	-	0.6 (0.4, 0.9)*	-	-	0.8 (0.6, 1.0)*	-	-	0.7 (0.5, 1.1)
**≥4 ^th^**	-	-	-	-	-	-	0.7 (0.4, 1.1)	-	-	0.7 (0.5, 0.9)*	-	-	0.6 (0.4, 0.8)*
**Place of** **delivery**	2.3 (1.6, 3.4)*	2.0 (1.5, 2.7)*	2.0 (1.4, 2.8)*	-	-	1.9 (1.3, 2.6)*	-	-	-	1.5 (1.2, 1.9)*	-	-	-
**Has a** **vaccine card**	3.3 (2.6, 4.1)*	7.8 (5.7,10.5)*	12.8 (7.9,20.7)*	10.8 (6.2,18.9)*	-	11.3 (7.5,17.0)*	7.6 (5.3,11.0)*	3.2 (2.4, 4.3)*	5.8 (4.5, 7.4)*	9.9 (7.9,12.4)*	9.1 (7.0,11.8)*	4.1 (3.0, 5.5)*	13.8 (10.0,19.0)*
**Rural** **residence**	-	-	0.6 (0.4, 0.9)*	2.0 (1.3, 3.1)*	-	-	-	-	-	-	-	-	-
**Mother's** **Education**												
**None**	-	-	Ref	-	-	-	Ref	-	-	-	Ref	-	-
**Primary**	-	-	1.8 (1.2, 2.7)*	-	-	-	0.9 (0.6, 1.3)	-	-	-	1.1 (0.8, 1.5)	-	-
**Secondary/** **higher**	-	-	3.1 (1.8, 5.4)*	-	-	-	2.3 (1.4, 3.7)*	-	-	-	1.9 (1.2, 2.9)*	-	-
**Mother's** **marital** **status**												
**Never** **married**	-	-	-	-	Ref	-	-	-	-	-	Ref	-	-
**Currently** **married**	-	-	-	-	2.2 (1.0, 4.7)*	-	-	-	-	-	0.5 (0.3, 0.8)*	-	-
**Formerly** **married**	-	-	-	-	9.7 (1.1,82.9)*	-	-	-	-	-	0.3 (0.1, 0.8)*	-	-
**Distance** **to health** **facility**	-	-	-	-	-	-	-	0.6 (0.4,0.8)*	0.8 (0.6,1.0)*	-	-	-	-
**Mother's age** **group**												
**≤24**	-	-	-	-	-	-	-	-	-	Ref	-	-	-
**25–29**	-	-	-	-	-	-	-	-	-	1.6 (1.2, 2.1)*	-	-	-
**≥30**	-	-	-	-	-	-	-	-	-	1.8 (1.3, 2.4)*	-	-	-

*Identifies the significant factors -Data omitted from this table because no association was seen in univariate analyses Ref – Reference for the various categories in each variable with >2 outcomes

Possession of a vaccine card was also significantly associated with full immunisation in all countries, except in Ghana, and the effect size was particularly significant in Togo where those with a vaccine card were almost 14 times more likely to be fully immunised.

Children who attended a check-up/clinic appointment within two months of birth had significantly greater odds of a FIC status in nine countries: Benin, Burkina Faso, Cote d’Ivoire, Guinea, Mali, Niger, Nigeria, Senegal and Togo.

In Liberia, Nigeria, and Togo, birth order was an important predictor of FIC. Compared to first-born children, second-born or higher birth order children had lower odds of FIC status only in Liberia, Nigeria, and Togo. Maternal education was also an important predictor of FIC in Cote d’Ivoire, Liberia, and Senegal, while the distance from vaccine clinics reduced the odds of FIC in Mali and Niger. The direction of the effects of socio-demographic factors like maternal marital status and rural residence on FIC status were not consistent across these West African countries. For example, rural residence was a predictor of FIC in The Gambia, but contributed to lower odds of full immunisation in Ivoirian children. Similarly, children born to mothers who were married or previously married were 2.2–9.7 times more likely to be fully immunised in Ghana, but this was 0.3–0.5 times lower in Senegal.

## Discussion

Despite the promising trend of increasing vaccination coverage observed in the region, the mean prevalence of the fully immunised child (FIC) was only 49%, indicating the poor state of vaccination coverage in this region and its constituent countries. Although there were significant inter-country variations in the prevalence of the FIC (ranging from 24% in Nigeria to 81% in Burkina Faso), the overall picture highlights the need for increased and consistent interventions to improve vaccination coverage in countries of West Africa. According to our analyses, some of the worst performing countries have made significant progress, but they are still clearly far off track towards achieving global vaccination coverage targets.

Although there was no single risk factor/predictor for FIC across all the West African countries covered in this study, delivery in a health facility and attending a well-baby clinic visit within two months of birth were common to over 50% of these countries. The other risk factors were less prevalent or relevant to fewer countries. In order to identify interventions to increase the proportion of the FIC in these countries, multidisciplinary investigations into the social (culture, religion, behaviour, etc.), health systems, political and economic correlates of childhood vaccination in countries of this region in much finer detail is essential.

In particular, this study highlights the enormous gap in the ability of healthcare service delivery system to minimize dropouts. The proportion of DPT1-to-DPT3 dropouts measure the consistency of a vaccination programme in delivering the same antigen(s) multiple times over a relatively short period. On the other hand, DPT1-to- MCV1 assesses dropouts over an extended period in the life of an infant and is considered a better measure of the overall effectiveness of immunization programs ^26^.

Children in Benin, Cote d’Ivoire, Guinea, Mali, and Nigeria were less likely to complete the schedule for repeated vaccines given DPT1-to-DPT3 dropout levels were higher than the WHO 10% threshold ^27, 28^. The reasons for this are not immediately obvious. All five countries are ranked quite low on the Human Development Index (HDI) with Mali and Guinea among the ten countries with the lowest HDI in Africa ( http://www.instituto-camoes.pt/images/cooperacao/relatorio_ocde14b.pdf). However, the conditions of literacy, life expectancy and income per capital resulting in a low HDI rank are only a part of the explanation, since Burkina Faso has excellent access and utilization of vaccination services and is also among the 10 lowest HDI ranked countries in Africa. Health system weaknesses caused by security challenges, civil war, insurgency, and political unrest are also very likely contributors. For example, Cote d’Ivoire suffered a second civil war in 2011, Guinea and Mali experienced health system weaknesses driven by political instability and insurgency, which for Guinea in particular, contributed to the country being the epicentre of the 2014 Ebola Epidemic. Mali’s predominantly rural population, a significant proportion that is nomadic scattered across a vast landmass that is the seventh largest in Africa, highlights its challenge with access and utilization of immunisation services. That Nigeria is the only country in Africa to have never eradicated polio perhaps underscores the challenges faced in delivery and access to vaccines, especially in the northern part of that country.

Only three countries -Ghana, Gambia and Burkina Faso - had acceptable DPT1-to-DPT3 drop-out levels i.e. <10%. The Gambia and Burkina Faso have good access to and utilization of immunization services despite a HDI rank of 175 and 185, respectively, suggesting other context specific issues contribute to high childhood vaccination coverage. Ghana ranked over 35 places on the HDI than Gambia, which is a country where >60% of the population live within a convenient distance from a health centre, and 80% vaccine coverage was achieved since 1990 ^29^. Unsurprisingly, the same countries with low DPT1-to-DPT3 dropout also had the lowest DPT1-to-MCV1 dropout, with the surprise inclusion of Benin.

Our findings here are similar to results found in other studies using dropout between BCG and MCV1 ^30, 31^. Since all, except the aforementioned countries, had DPT1-to-MCV1 dropout of >10%, there is clearly a region-wide problem with retention of infant cohorts by immunisation services. With the Ebola-hit countries, Liberia, Guinea, and Sierra Leone, which were among those with the highest dropout, poor retention confirms that a weak health system is a predisposing factor to disease outbreaks and significant mortality from VPDs ^32, 33^. There are large inter-country differences observed in access (DPT1-to-DPT3 drop-out) and utilization (DPT1-to-MCV1 drop-out) of immunization programmes. In order to identify and implement interventions to increase access and utilization, affected countries need to conduct in-depth investigations of associated risk factors/predictors for poor vaccine coverage and healthcare utilization ^34^.

In all countries, except Ghana, having a vaccine card was a significant predictor of FIC. Vaccine cards are thought to be essential in promoting complete vaccination in children by acting as information resources ^16^. Similar to our findings, other studies have also shown the importance of vaccine cards in promoting child health and immunization ^13, 35^.

Delivery in a hospital or a health facility is associated with higher vaccine coverage because early contact with the healthcare system during and following parturition ensures prompt delivery of birth vaccines (BCG, OPV0, and Hepatitis B) and provides an opportunity to reinforce the need for immunization to mothers and other caregivers ^7, 21^. Similarly, increasing contact time with the healthcare system through post-natal appointments has also been associated with increased vaccination coverage. As shown in this study, receiving a check-up within two months of birth was associated with full immunisation. These kinds of visits achieve this by affording health care workers opportunities to review vaccination histories, and reinforce (and/or clarify) vaccine-related information. It also results in reduced missed opportunities and contributes to higher compliance with the childhood immunisation schedule ^7, 36^.

It is not surprising that, along with higher maternal age (one country), maternal education (three countries) was associated with complete vaccination status. Other investigators have reported educated mothers tend to have better health care seeking behaviour, due to a better understanding of the benefits of medical care, including the benefits of spacing their children ^37^.

Our study had some limitations. We used DHS data that is designed to be comparative at national and regional levels, so the findings here do not have the richness of detail for a finer examination of coverage and associated risk factors at the level of districts or settlements. These surveys assess vaccination status by vaccine card record and parental recall. Recall of vaccination history introduces recall bias. In addition, using vaccine cards as a source document from which vaccination histories were obtained alongside recall may have introduced some ascertainment bias. It is possible the varying effect sizes for vaccine cards observed was driven by card retention within countries and the contribution of parental recall to the ascertainment of exposure to vaccination, which may overestimate vaccination coverage.

Even with a probability-based sampling design, underserved populations or those most likely to be unimmunized may be undersampled by vaccination coverage surveys, like DHS. Clusters with a larger population are more likely to be selected compared to less populated clusters, increasing the risk of oversampling high populated areas and making the sample less representative. The net effect of this will be exaggerated vaccination coverage. To counter this, we applied survey weighting in our analyses.

It would have been useful to examine other sources of survey coverage data to validate or triangulate the DHS data used in the study. One likely source is from the results of the UNICEF-led Multiple Indicator Cluster Surveys (MICS, http://mics.unicef.org/surveys). However, this was not possible for two key reasons. First, the survey years do not overlap, and secondly, there is a slight difference in methodology as DHS surveys record all vaccines given before the survey, while MICS looks at all vaccines given just before the first birthday in the target population. Nonetheless, the DHS surveys are rigorously designed and utilize well validated methodology.

## Conclusions

Most West African countries lag significantly behind the global targets of 90% national vaccination coverage and < 10% dropout between vaccine doses. Although there has been some progress in increasing vaccination coverage in the region, global and regional vaccination coverage targets will not be achieved without improving on and maintaining the uptake of vaccines in these countries. Health system weaknesses and inefficiencies responsible for the unacceptably high dropout rates between vaccine doses needs to be tackled urgently. Given the importance of the correlates of complete vaccination in the region, as highlighted in this study, countries should implement interventions to address locally relevant predictors of complete vaccination to achieve and sustain higher vaccination coverage, leading towards the end of preventable deaths of newborns and children less than five years of age by 2030.

## Data availability

The datasets and variable codebooks are freely and publicly available on request from the DHS Program website, http://dhsprogram.com/What-We-Do/Survey-Types/DHS.cfm (searching by country is the easiest way to access the datasets used in the current study: http://dhsprogram.com/What-We-Do/survey-search.cfm?pgtype=main&SrvyTp=country). We have therefore only made available the data cleaning and analysis code used in generating the findings reported in our publication on the Invasive Bacterial Disease Research Group Dataverse: doi, 10.7910/DVN/YQYES9 ^38^.
