# Supplementary material for: Crude childhood vaccination coverage in West Africa: Trends and predictors of completeness
Source: Wellcome Open Res. 2017 Feb 15;2:12. [Version 1] doi: 10.12688/wellcomeopenres.10690.1 (PMC5407439; doi:10.12688/wellcomeopenres.10690.1)
Supplement: Peer review history

# Peer review: Referee response for version 1

**DOI:** 10.21956/wellcomeopenres.11526.r20713

**Published:** 2017-04-18

**Authors:** Birger C. Forsberg

**Affiliations:** 1 Department of Public Health Sciences, Karolinska Institute, Solna, Sweden

A well written article with interesting results, though mostly known already, that are most important for policy making. Some comments for further improvements: 

1. "explored" in the Abstract is in past tense while the first verb ("review") is in present tense. Chose either. 
2. In the "Background" section it is stated that "Most vaccination coverage estimates in LMICs are from administrative reports that tend to overestimate coverage, due to errors in the number of vaccine doses administered and/or invalid assumptions about the size of the target population of children." This statement should by preference be supported by a reference. I would say that most estimates are based on surveys while vaccine coverage reports may be based on administrative reports, i.e. data from routine reporting in the health system.
3. Under "Methods" the risk factors considered are listed. However, the factors listed should rather be called "determinants". For instance, maternal education is not a risk factor in itself but a determinant. Low maternal education carries a risk for  lower participation in vaccine programmes. The opposite goes for high education.
4. As pointed out by other referees, it is questionable if "possession of vaccine card" can be put in the logistic regression model if outcome (children vaccinated) is measured with vaccine card. Clearly, there is a risk that possession of vaccine card increases your chance of being included in the sample.
5. Under "Ethic statement" it is said that "...written informed consent is obtained from each survey participant" and a reference to DHS is given. I would soften this fact and make it clearer that it is coming from the DHS report. It could for instance be formulated "The DHS programme reports that....".
6. There are two "after" after each other in the sentence following the one quoted under (5).
7. On page 7 in the second paragraph in the section titled "Predictors of complete vaccination status" the term risk factor is again used in an inaccurate way, I believe. A "risk factor" is related to a negative outcome (in order for it to be a "risk"). Hence, there cannot be "risk factors for complete vaccination". They should be called "determinants" of "predictors".
8. The article is based on an analysis between countries under the standard assumption that countries form relatively coherent entities suitable for comparison. However, intra-country variation may be as important as inter-country variation when it comes to primary care services. Hard to reach regions may be more or less represented in the countries compared. It would be useful if the authors could comment on this somewhere in the article, preferably in the discussion section.
9. Under "Discussion" the first sentence states "...the mean prevalence of the fully immunized child (FIC) was only 49%, indicating the poor state.....". No numbers on aggregated "means" for all countries are given anywhere in the Results section. It is therefore surprising that it appears in the Discussion section. It is not described how the authors calculated this mean prevalence. I suggest the sentence is taken out as it opens up to a different data analysis than the one used in the article (where data from each country are presented separately and compared.)
10. On top of page "capital" should be "capita".
11. In the second column, it is said in the fourth paragraph (starting "Even with a probability...) that "Clusters with a larger population are more likely to be selected compared to less populated clusters..:" This is not correct. The cluster sampling is not a method in which you select your sample from clusters. It is a method through which you select clusters from a population enumeration based on an estimate or  census data. All persons in the population have the same chance of being included in a cluster, irrespective of there they live. In EPI coverage surveys a cluster typically consists of 7 children.
12. In the "Conclusions" section it is said that "Health system weaknesses and inefficiencies responsible for the unacceptably high dropout rates between vaccine doses need to be tackled urgently." However true this may be, there is in fact little to no evidence in the article that health system weaknesses and inefficiencies are responsible for the drop out rates documented in the surveys. The reason is that this variable is not included in the regression model. It would be possible, but not easy and uncontroversial, to include a proxy for such system weaknesses in the model but rather than redoing their analysis, I suggest the authors move the quoted sentence to the "Discussion" section and then qualify their statement. The "Conclusion" section should focus on the actual results from the study and their policy implications.      

I have read this submission. I believe that I have an appropriate level of expertise to confirm that it is of an acceptable scientific standard.

# Peer review: Referee response for version 1

**DOI:** 10.21956/wellcomeopenres.11526.r21160

**Published:** 2017-04-04

**Authors:** M Carolina Danovaro-Holliday

**Affiliations:** 1 Expanded Programme on Immunization, Department of Immunization, Vaccines and Biologicals, World Health Organization, Geneva, Switzerland

Crude childhood vaccination coverage in West Africa: Trends and predictors of completeness

 This manuscript presents analyses of DSH from 13 West African countries in terms of fully immunized child or FIC (defined as having received basic vaccines) and drop-out, along with trends between two DHS surveys for 10 of these countries. It is well-written and presents a useful picture of the situation of a group of countries and highlights that there is much work to do in terms of improving coverage and reducing drop-outs. It also shows that the factors associated with FIC differ among countries.

 The main issue I have with the study is that it only presents coverage point estimates. It fails to show the % of cards seen and confidence intervals around estimates. This limits data interpretation. Also, at least to me, it is not clear if the authors were able to replicate the results included in the respective DHS report. If it was a re-analysis, it is frequent that estimates are a bit different due to non-explicit decisions on record or data point exclusion, or how to treat “don’t know” responses, for example. This is not mentioned.

 Also, by calling the drop-out DTP1-DTP3 an “access” issue and the drop-out DTP1-MCV1 a “utilization” issue, it creates confusion, as often DTP1 coverage is used to denote access and drop-out (any) to denote utilization. See: Immunization in Practice. Module 6. Monitoring and Surveillance http://apps.who.int/iris/bitstream/10665/193412/1/9789241549097_eng.pdf

 Regarding the exclusion of MICS surveys, at least the most recent MICS do include vaccination history not limited to those received before their first birthday. See: http://mics.unicef.org/tools and Cutts *et al.*, 2013.

 Finally, though the results illustrate a group of countries in a sub-region, generalizing to “West Africa” seems an unneeded stretch.

 Specific comments:

 Abstract

- Provide a timeframe, e.g., from 2003 DHS in Burkina Faso and Ghana to 2013.

Introduction

- Review the references for the statement about drop-out. While the webpages cited do include a recommendation on attaining coverage >=90% at the national level, they do not include a target for drop-out. The 10% is commonly used, but the reference needs to be added. It could be Immunization in Practice. Module 6. Monitoring and Surveillance http://apps.who.int/iris/bitstream/10665/193412/1/9789241549097_eng.pdf
- Add a reference for the statement about admin reports most frequently overestimating coverage
- Consider Lim *et al.*, in addition to, or instead of, reference 11
- You may want to add here why you didn’t include MICS

Methods

- Consider changing to univariate to bivariate

Results

- Present uncertainty data
- Include the % with cards seen
- Table 3 is confusing. It could add coverage “before” and “after” to better understand what the % presented is about
- Remove data interpretation when presenting drop-out results, as the definitions were included in the methods

Discussion

- Avoid presenting a summary figure
- Again, revise the references used when presenting a threshold of 10% for drop-out
- Discuss the potential use of cards as an explanatory variable, given that vaccination ascertainment may be affected by this
- Revise the use of access and utilization when referring to drop-outs
- Consider adding the limitation that surveys do not directly obtain data on barriers to vaccination
- Consider adding the limitation that untimely vaccination may occur. See a recent review “Factors associated with incomplete or delayed vaccination across countries: A systematic review” by Tauil *et al.*, Vaccine 2016.

Conclusions

- I don't think the data presented allows determining the cause of the mostly low coverage and high-drop outs found. Thus, I suggest removing or rewording “Health system weaknesses….to be tackled urgently”. It may be true, but it is not a conclusion based on the data presented.

References

- Consider further references by Brown *et al.* on home-based records, more on timeliness and references on the Global Vaccine Action Plan (GVAP), see: http://www.who.int/immunization/global_vaccine_action_plan/GVAP_doc_2011_2020/en/

I have read this submission. I believe that I have an appropriate level of expertise to confirm that it is of an acceptable scientific standard, however I have significant reservations, as outlined above.

# Peer review: Referee response for version 1

**DOI:** 10.21956/wellcomeopenres.11526.r20250

**Published:** 2017-03-03

**Authors:** Charles S. Wiysonge, Abdu Adamu

**Affiliations:** 1 Cochrane South Africa, South African Medical Research Council, Cape Town, South Africa

**General comments**

 We commend the authors for choosing a very topical issue for the manuscript. However, we have concerns with the amalgamation of data from different years in different countries. For example, the authors combine 2008 data from Ghana and 2014 data from Togo, to give an overall estimate of vaccination coverage in West Africa. To which year did the authors assign the 49% mean vaccination coverage for West Africa? In the same vein, if the authors were asked to assess whether West Africa achieved the GVAP coverage target for 2015 (using the two countries mentioned above as examples), would they be comfortable combining 2009 data for Ghana and 2015 coverage data for Togo to give 2015 figures for the region?

**Specific comments** 

- In the abstract, the authors should mention the years for the coverage data.
- We do not think that the average coverage reported for West Africa is informative, for the reason stated above.
- For the average coverage data, the median and range would be more informative than mean and range.
- The authors refer to “…a trend of increasing vaccination coverage…” in the abstract. If this trend is over time, would it not be helpful to indicate the years of interest?
- We found it difficult to understand the concept of “risk factor for full immunization coverage” in this study. Could there be a more informative phrase than “risk factor” in this context? Is maternal education a risk factor for vaccination in a similar sense to smoking being a risk for lung cancer?
- Given that vaccination coverage was assessed from records in vaccination cards, we doubt whether it is appropriate to put “possession of a vaccination card” in the multivariate logistic regression model.
- It would be helpful in the abstract, to provide the effect estimates and their 95% confidence intervals for the respective effects on vaccination coverage of maternal education, delivery in a health facility, etc.
- The authors report “Chi-square tests were utilized in univariate analyses to examine associations between FIC and possible risk factors”. Should the analyses be referred to as “bivariate”, given that the authors examined the relationship between two variables?

Overall, we read the manuscript with great interest and appreciate the efforts of the authors.

We have read this submission. We believe that we have an appropriate level of expertise to confirm that it is of an acceptable scientific standard, however we have significant reservations, as outlined above.

# Peer review: Referee response for version 1

**DOI:** 10.21956/wellcomeopenres.11526.r20251

**Published:** 2017-02-27

**Authors:** Lars T. Fadnes

**Affiliations:** 1 Department of Global Public Health and Primary Care, University of Bergen, Bergen, Norway

I think the study is generally well written and well presented. The analyses are generally appropriate; however there are a few details that need to be checked and clarified. 

**Major revision:**

 When vaccination cards were used to obtain immunization information, can they then be assessed as a factor in a multivariable regression analysis on whether they were fully vaccinated (particularly as parent recall of individual vaccines is challenging)? 

 Nevertheless, for the point of the discussion and independent of this paper, there are good reasons to believe vaccination cards are of importance for obtaining appropriate and timely immunization.

**Minor revisions:**

 Timeliness of vaccination can also impact on vaccine efficacy. Please add a section on timeliness of vaccination in the discussion.

I have read this submission. I believe that I have an appropriate level of expertise to confirm that it is of an acceptable scientific standard, however I have significant reservations, as outlined above.
